# Biogenic Silver Nanoparticles: What We Know and What Do We Need to Know?

**DOI:** 10.3390/nano11112901

**Published:** 2021-10-29

**Authors:** Mahendra Rai, Avinash P. Ingle, Joanna Trzcińska-Wencel, Magdalena Wypij, Shital Bonde, Alka Yadav, Gabriela Kratošová, Patrycja Golińska

**Affiliations:** 1 Faculty of Biological and Veterinary Sciences, Nicolaus Copernicus University in Toruń, Lwowska 1, 87-100 Toruń, Poland; trzcinska@doktorant.umk.pl (J.T.-W.); mwypij@umk.pl (M.W.); 2 Department of Biotechnology, Sant Gadge Baba Amravati University, Amravati 444602, India; shitalbonde@gmail.com (S.B.); nanoalka@gmail.com (A.Y.); 3 Biotechnology Centre, Department of Agricultural Botany, Dr. Panjabrao Deshmukh Krishi Vidyapeeth, Akola 444104, India; ingleavinash14@gmail.com; 4 Nanotechnology Centre, CEET, VŠB–Technical University of Ostrava, 17. listopadu 2172/15, 708 00 Ostrava Poruba, Czech Republic; gabriela.kratosova@vsb.cz

**Keywords:** silver nanoparticles, biosynthesis, application, biodistribution, toxicity

## Abstract

Nanobiotechnology is considered to be one of the fastest emerging fields. It is still a relatively new and exciting area of research with considerable potential for development. Among the inorganic nanomaterials, biogenically synthesized silver nanoparticles (bio-AgNPs) have been frequently used due to their unique physicochemical properties that result not only from their shape and size but also from surface coatings of natural origin. These properties determine antibacterial, antifungal, antiprotozoal, anticancer, anti-inflammatory, and many more activities of bio-AgNPs. This review provides the current state of knowledge on the methods and mechanisms of biogenic synthesis of silver nanoparticles as well as their potential applications in different fields such as medicine, food, agriculture, and industries.

## 1. Introduction

Nanotechnology is emerging as a science of the 21st century that has attracted a great deal of attention from the global scientific community due to its pathbreaking innovations and applications in varied sectors [1]. Nanomaterials are considered to be the building blocks of nanotechnology and are found to possess various unique properties, such as optical, mechanical, catalytic, and biological properties, etc., that make them the materials of choice for nanotechnologists. In addition, features such as the extremely small size of nanomaterials, their high surface area-to-volume ratio, high reactivity, and compatibility, etc., enhance their suitability for various purposes, including biomedical applications [2]. To date, different physical, chemical, and biological approaches have been proposed for the synthesis of nanoparticles. Among these approaches, physical and chemical approaches are reported to have control on the size of synthesized nanoparticles, but they have their own shortcomings. As far as physical synthesis is concerned, it involves usage of radiation (e.g., microwave, UV light, etc.), high temperature, and pressure, which might be hazardous. Moreover, in the case of chemical synthesis, usage of toxic chemicals is the main drawback. Considering these issues, the demand for a newer, novel, and ecofriendly synthesis approach was raised, and that search has been fulfilled with the option of biological synthesis of nanoparticles [3,4,5,6].

Recently, the green synthesis of nanomaterials has garnered the attention of nanotechnologists and has given birth to a new branch known as “green nanotechnology” [7]. The biosynthesis of AgNPs is considered to be a green route as it follows the principles of green chemistry and is the basis of sustainable development. It is usually performed by using various microorganisms such as fungi, bacteria, green algae, and also plants. Biosynthesis has several advantages over the physical and chemical processes because it is eco-friendly, cost-effective, and can be executed at ambient temperature and pressure, with enhanced bioactivity, and less toxicity. It was demonstrated that the proteins and enzymes secreted by the above-mentioned bio-systems act as reducing agents that reduce the bulk metal salt into respective nanoparticles and also act as capping agents that provide stability to nanoparticles and make them bio-compatible for various biological applications [8,9]. Among the metal nanoparticles, silver nanoparticles (AgNPs) have been revealed to have an enormous number of applications, particularly in biomedicine due to their broad range of antimicrobial potential, including antibacterial, antifungal, antiviral, and antiprotozoal properties [10,11]. Therefore, special focus has been given to biogenic AgNPs.

In this review, we discuss different methods of synthesis of AgNPs, with special reference to bio-inspired synthesis and purification of nanoparticles. The different views on the mechanism involved in biosynthesis provide insights into how AgNPs are synthesized by biological systems. We also elaborate the applications of AgNPs in medicine, agriculture, textiles, sensors, etc. The biodistribution and toxicity of biogenically synthesized AgNPs are also discussed.

## 2. Biosynthesis Is a Solely Green and Sustainable Technology for AgNPs Synthesis

Metal nanoparticles are well known for their unique physical, chemical, and biological properties, which depend on their size, shape, and surface charge [12,13]. The morphology and dispersity of metal nanoparticles depend on the mode of their synthesis [14]. Mostly the synthesis of nanoparticles is based on physical and chemical methods; however, the current studies in this field has raised concerns regarding the possible risks and the related effects of nanoparticles on the environment and humans [15]. Due to the growing application of nanoparticles in almost every field of science and technology, researchers have emphasized the biological synthesis of metal nanoparticles [14]. Among the different nanoparticles studied to date, AgNPs have achieved a significant place due to their excellent antimicrobial property against a broad range of pathogenic micro-organisms [16].

**Figure 1:**
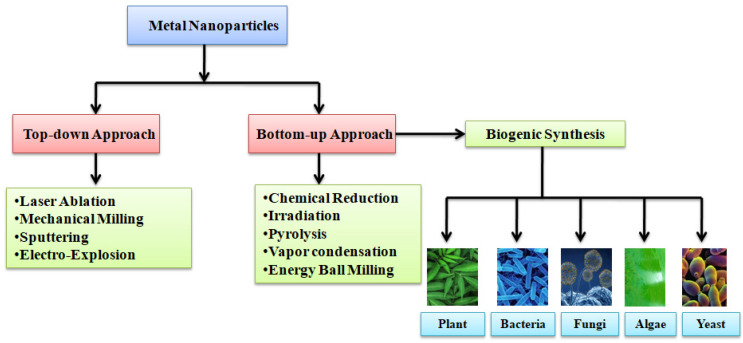
Synthesis of metal nanoparticles.

The biosynthesis of AgNPs using bacteria, fungi, yeasts, algae, and plants has garnered much attention in the recent decade [13]. Biosynthesis is the process of utilization of plant extracts, different microbes, and enzymes for the synthesis of metal nanoparticles (Figure 1). This process offers cost-efficient, environment-friendly, and scalable fabrication of nanoparticles without the use of any toxic chemicals [12]. Furthermore, the synthesis process can be sub-divided into two types i.e., a top-down and a bottom-up approach [15,16].

### 2.1. Strategies for Synthesis

**Figure 2:**
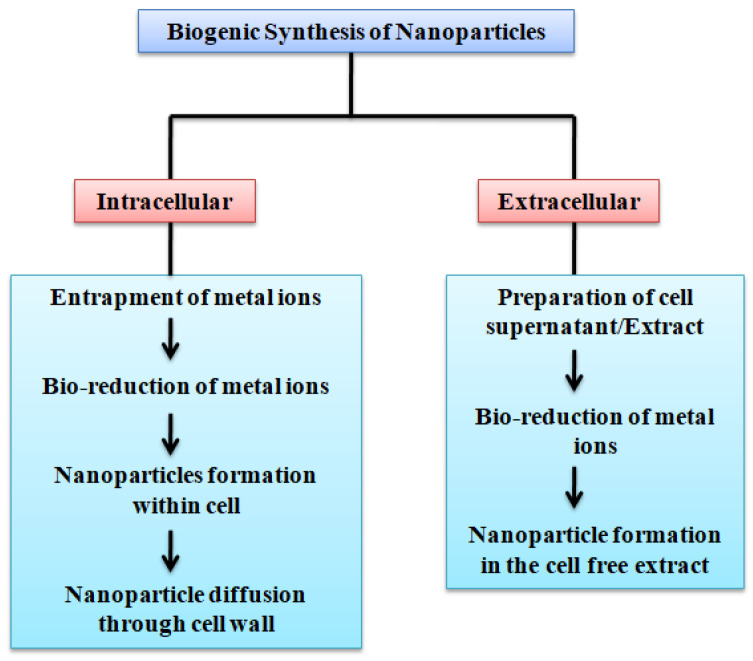
Methods of biosynthesis of metal nanoparticles.

AgNPs are synthesized using physical, chemical, and biological methods. The chemical synthesis of AgNPs is achieved through chemical reduction, the electrochemical method, irradiation, and pyrolysis [15]. For the chemical synthesis process, reducing and stabilizing agents are required. Generally ascorbic acid, ethanol, borohydride, sodium citrate, etc. are used as a reducing agents [14]. During the chemical and physical synthesis use of hazardous chemicals, the high temperature and pressure needed to perform the synthesis of nanoparticles are a matter of great environmental concern [16]. Table 1 summarizes important physical, chemical, and photochemical methods commonly used for the synthesis of AgNPs. Based on the aforementioned problems of chemical and physical synthesis methods, biological synthesis appears to be the most acceptable method in terms of environmental impact. Biological synthesis involves the fabrication of AgNPs using micro-organisms or plant extract, which signifies an environmentally friendly approach towards the synthesis of metal nanoparticles. It also shows several advantages over the chemical and physical methods because of its simple, cost-efficient, high-yielding nature [15]. Biological synthesis can further be sub-divided into extracellular synthesis and intracellular synthesis. In the intracellular synthesis method, nanoparticles are synthesized inside the cells while, in extracellular synthesis, cell-free fungal extract is used (Figure 2).

### 2.2. Potential Biological Agents for Eco-Friendly Synthesis of AgNPs

**Figure 3:**
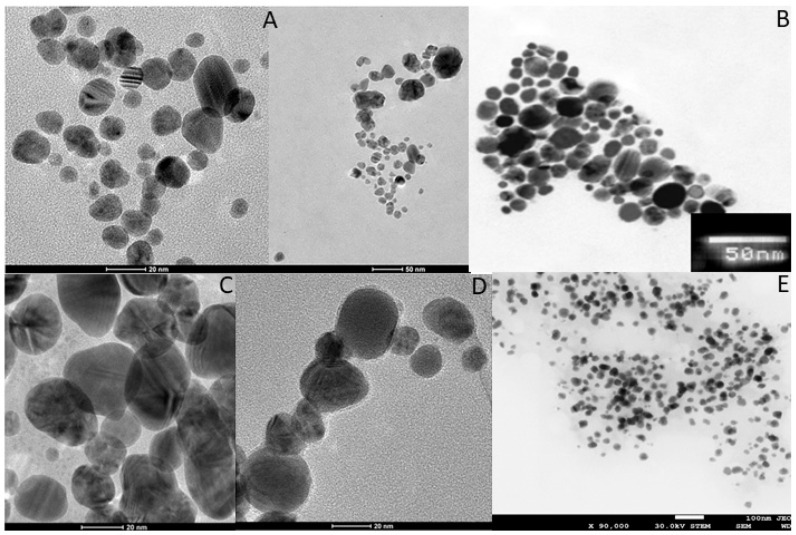
TEM micrographs of bio-AgNPs from (**A**) actinobacterial strain SF23 (adapted from [9], open access article), (**B**) myxobacteria, (**C**) *Fusarium oxysporum*, (**D**) *Fusarium tricinctum*, and (**E**) *Urtica dioica*.

Green synthesis of nanoparticles is accomplished by biological agents including bacteria, fungi, algae, yeasts, and plant extracts. It employs the use of a suitable solvent system and biological agents to achieve synthesis of nanoparticles [13]. Different microorganisms such as bacteria, fungi, yeasts, algae, and plants used for biogenic synthesis of AgNPs are shown in Table 2. Many bacteria such as *Bacillus siamensis* [17], *Shewanella* sp. ARY1 [18], *Citrobacter freundii* [19], and cyanobacteria *Leptolyngbya* sp. WUC 59 [20] have been used for the synthesis of AgNPs. Similarly, various fungi including *Aspergillus flavus* [21], *Trichoderma harzianum* [22], *Talaromyces purpurogenus* [23], and *Fusarium scirpi* [24] have been used for the biogenic synthesis of AgNPs. Plant extracts obtained from *Protium serratum* [25], *Zea mays* [26], *Eucalyptus corymbia* [27], and *Lysiloma acapulsensis* [28] have been recently harnessed for biogenic synthesis of AgNPs. Apart from the aforementioned bacteria, fungi, and plant species, several other biological agents have been exploited for the synthesis of AgNPs, e.g., food and agricultural wastes [29]. Figure 3 shows the TEM micrographs of AgNPs synthesized by different biological agents.

### 2.3. Fungi as Promising Myconanofactories

Mycosynthesis is used for the production of metal nanoparticles using fungi [40]. Interestingly, the fungal extracts show high tolerance to metals, and the biomass is easy to control. The biomolecules present in the fungal extracts enable easy reduction of metal ions and also ensure the stability of the nanoparticles [41]. The fungal system provides a single step biosynthesis of AgNPs and can be used for both intracellular and extracellular biosynthesis [21]. The mycelial mass of fungi can sustain a higher amount of agitation and pressure, and hence can be used for large scale biogenic synthesis [35]. The change in external parameters such as pH, temperature, light, and amount of biomass influences the size and morphology of the AgNPs [40].

For the intracellular synthesis of AgNPs using fungi, metal salt solution is added to the fungal culture and subsequently after synthesis of AgNPs, centrifugation and filtration is accomplished to disrupt mycelial biomass and AgNPs [41], while in the extracellular synthesis of AgNPs by fungi, silver salt solution is added to the aqueous cell-free fungal filtrate, which results in simple and fast synthesis of AgNPs due to the presence of fungal enzymes. Extracellular synthesis of AgNPs using fungi has been widely followed [40]. Further, the AgNPs dispersion is purified to remove fungal contaminants through filtration, dialysis, and ultracentrifugation [21,41].

Thus, the fungal system has an advantage over the bacterial and plant system owing to the presence of a large amount of extracellular protein in the filtrate, which enables large-scale synthesis of AgNPs, and also the downstream processing of fungal biomass is much simpler compared with the other systems.

### 2.4. Microfluidic Approach in Nanoparticles Biosynthesis

Microfluidics is the science and technology of manipulating and controlling fluids, usually in the range of microliters (10^−6^) to picoliters (10^−12^), in networks of channels with the lowest dimensions from tens to hundreds of micrometers [42]. It is a multidisciplinary field that brings together physics, chemistry, engineering, biochemistry, and also nanotechnology. Microfluidic systems are preferably used in the pharmaceutical industry [43], but there are already a number of publications that demonstrate the advantages of synthesis of nanoparticles by microfluidics [44,45]. Generally known benefits of controlled flow synthesis on a microfluidic chip compared with batch synthesis are precise control of reaction, high conversion and selectivity, better reaction yields, flexibility, safety, uniform product quality, contamination prevention, and lower consumption of reactants. Therefore, it is not surprising that microfluidics coupled with nanotechnology begins to complement the synthesis of nanoparticles by lab-on-a-chip systems. The use of microfluidic techniques and the implementation of micro-reactors in production processes in nanotechnologies can optimize both conventional chemical and biotechnological bottom-up approaches.

Because of some issues of batch biosynthesis—typically process reproducibility and nanoparticles heterogeneity in terms of their shape and size—the scientific community is currently focusing on nanoparticles phytosynthesis using microfluidic microreactors with various channels geometry made from different materials [46,47]. The main advantage of low-cost biosynthesis, i.e., reduction and simultaneous stabilization of AgNPs, is thus enhanced by controlling the flow process. Recently, the design of reactors and their production using 3D printers has also been preferred. In general, applying microfluidics with completely different results regarding shape, size, and stability of metallic nanoparticles may be achieved, as compared to the batch synthesis. However, various process conditions must be studied to find the one that is optimal for a given plant biomass, metal precursor, and micro-reactor.

## 3. Unzipping the Mechanism Involved in Biogenic Synthesis of AgNPs

**Figure 4:**
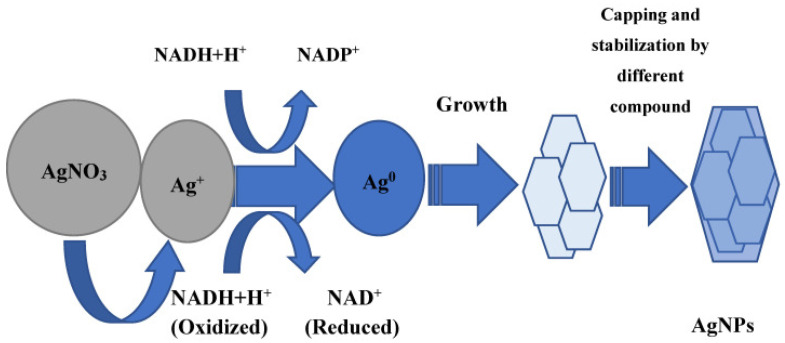
The general hypothetical mechanism proposed for the mycosynthesis of AgNPs (adapted from [55]; an open access article).

Plants, fungi, bacteria, cyanobacteria, blue-green algae, and actinomycetes are the most important biological systems used for the synthesis of AgNPs [39,48,49]. There are different mechanisms put forward by the researchers to explain the biogenic synthesis of nanoparticles based on their research outcomes [50,51,52,53,54]. However, the most widely studied mechanism for biosynthesis of any metal nanoparticle involves the reduction of aqueous metal ions by the donation of an electron from a particular compound or biomolecule that is present in the extracellular metabolites of the extract of the biological system. These compounds play an important role in transferring electrons to aqueous metal ions to fulfill the deficiency and reduced to their neutral form, which is commonly referred to as a nano form or nanoparticles (Figure 4). In some cases, the involvement of more than one or group of biomolecules is reported to be involved in the biosynthesis and stabilization of AgNPs. Some of the important and widely accepted mechanisms are discussed here.

### 3.1. Mechanism Involved in Bacterial Synthesis of AgNPs

Different mechanisms have been suggested for the bacterial synthesis of nanoparticles. Bacteria also have potential to synthesize metal nanoparticles both intracellularly and extracellularly. The available reports suggest that extracellular synthesis of AgNPs is typical for both Gram-positive bacteria (e.g., *Bacillus pumilus*, *B. persicus*, *B. licheniformis*, *B. indicus, B. cecembensis*, *Planomicrobium* sp., *Streptomyces* sp., *Rhodococcus* sp.) and Gram-negative bacteria (e.g., *Klebsiella pneumoniae*, *Escherichia coli* and *Acinetobacter calcoaceticus*) [56,57,58,59,60,61,62]. Similarly, certain Gram-positive (e.g., *Corynebacterium* sp., members of the genus *Streptomyces*) [63] and Gram-negative bacteria such as *Enterobacter* and *Pseudomonas stutzeri* have been reported to produce AgNPs intracellularly [64,65]. As far as the mechanism in intracellular synthesis of AgNPs is concerned, it was proposed that membrane proteins are mainly responsible for the transport of silver ions into the bacterial cells, where they are reduced by enzymes and other metabolites [66]. The studies demonstrated that proteins and sugars of the cell wall (where the actual reduction process can occur) also participate in the catching of the silver ions [55].

The entrapment and transportation of silver ions in bacterial cells may be facilitated by electrostatic interaction of the positively charged silver ions and negatively charged cell wall, particularly the negatively charged carboxylate groups present on the cell wall [67]. On the other hand, different metabolites such as NADH-dependent reductase (Figure 4), sulfur-containing protein, etc. secreted by the bacteria in the surrounding medium are mainly responsible for the extracellular reduction of silver ions into AgNPs [68,69]. In addition, different amino acids viz. arginine, aspartic acid, cysteine, glutamic acid, lysine, and methionine have the capability to reduce silver ions to AgNPs [70,71]. The possible role of some peptides containing disulfide bonds was also proposed in the reduction of silver ions to AgNPs [72].

According to one of the theories, the synthesis of metal nanoparticles by bacteria is the outcome of detoxification pathways, where a variety of toxic metal ions are taken up through cationic membrane transport systems that normally transport metabolically important cations. The specialized mechanism to counteract this kind of uptake prevents excessive accumulation of toxic metals [73].

### 3.2. Mechanism of Mycosynthesis of AgNPs

Generally, it is proposed that the extracellular metabolites, including different enzymes secreted by fungi for their own survival when exposed to different environmental stresses, are responsible for the reduction of metal ions to metallic solid nanoparticles through the catalytic effect [32,40]. In one of the mechanisms, it was proposed that NADH-dependent nitrate reductase enzyme secreted by *Fusarium oxysporum* is responsible for the reduction of aqueous silver ions into AgNPs [74]. A similar mechanism was proposed by Ingle et al. [75] in the case of AgNPs synthesis from *Fusarium acuminatum.* The authors also pointed out the involvement of cofactor NADH and nitrate reductase enzyme in the biosynthesis of AgNPs owing to the presence of nitrate reductase in fungal cell-free extract, which was proved by using specific substrate-utilizing discs for nitrate purchased from Hi-Media Pvt. Ltd., Mumbai, India. Finally, it was proposed that nitrate reductase secreted by the biological system is responsible for the reduction of silver ions into AgNPs, and NADPH acts as co-factor and helps in transferring the charge (electron) [74,75]. NADPH is well-known for its biological role as a coenzyme, mediating charge transfer between enzymes and its natural substrate (Figure 4). However, it was demonstrated that NADPH can independently act as a reducing agent in the case of charge transfer to an inorganic compound such as metal salts. In this context, Hietzschold et al. [53] studied the role of both NADPH and nitrate reductase enzyme in the biogenic synthesis of AgNPs. This study suggests that NADPH can serve as the sole reducing agent in reducing the silver ions to AgNPs. Moreover, it was revealed that for the synthesis of AgNPs, nitrate reductase is not required. Further, it was also found that AgNPs formed in the enzyme-free reaction were smaller, monodispersed, and more stable than those synthesized with the addition of nitrate reductase. However, more studies are required to arrive at a definitive conclusion.

**Figure 5:**
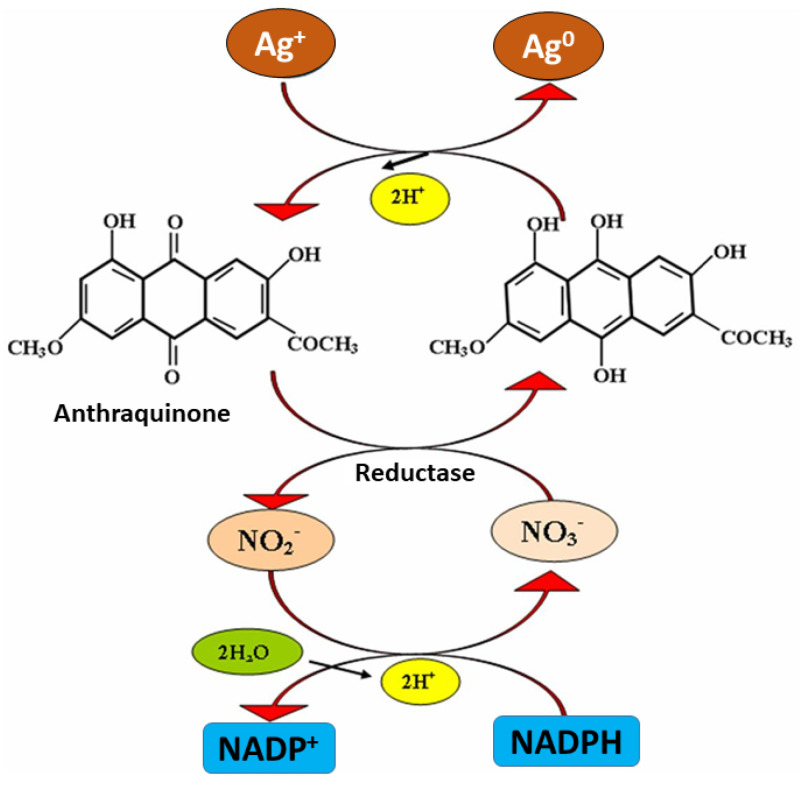
Possible anthraquinone-mediated hypothetical mechanisms for AgNPs synthesis (adapted and modified from [76]; an open access article).

In another studies, Durán et al. [76] and Kumar et al. [77] proposed almost similar mechanisms for the biosynthesis of AgNPs from *F. oxysporum*. The former study reported the role of anthraquinones and NADPH-nitrate reductase in the biosynthesis of AgNPs. The electron required to fulfill the deficiency of aqueous silver ions (Ag^+^) and convert it into Ag neutral (Ag^0^ i.e., AgNPs) was donated by both quinone and NADPH (Figure 5). However, in a later study, it was demonstrated that the reduction of NADPH to NADP^+^ and hydroxyquinoline possibly act as an electron shuttle by transferring the electron generated during the reduction of nitrate to Ag^+^ ions and finally converting them to Ag^0^. Moreover, Li et al. [78] also suggested a similar mechanism in the case of the synthesis of AgNPs by using *Aspergillus terreus*.

Apart from these, the role of proteins and amino acids having -SH groups such as cysteine is also confirmed in the biosynthesis of metal nanoparticles. It is proposed that such amino acid undergoes a dehydrogenation reaction with metal salts such as silver nitrate and produces AgNPs [79]. Moreover, various amide groups (I, II, and III) are also reported to play an important role in the biosynthesis of metal nanoparticles. Sanghi and Verma [80] reported the involvement of amide I and amide II groups in the synthesis of AgNPs from *Coriolus versicolor*. However, in case of xylotrophic fungi, it was demonstrated that its oxidation and reduction system depends on phenol oxidase enzymes, such as Mn-peroxidases, laccases, and tyrosinases. Therefore, it can be concluded that such enzymes may play a pivotal role in the biosynthesis of metal nanoparticles with fungal extracts [81].

**Figure 6:**
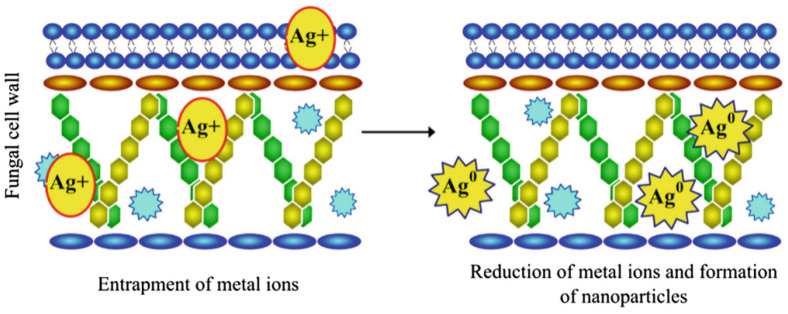
Possible hypothetical mechanism for intracellular mycosynthesis of AgNPs, reproduced with permission from Yadav et al. [40].

As far as the intracellular synthesis of AgNPs is concerned, it was proposed that metal nanoparticles are usually synthesized below the cell surface [82], and this reduction process is mainly governed by enzymes present in the cell membrane. In intracellular mycosynthesis, initially, the entrapment of metal ions occurs due to their electrostatic interaction with lysine residues on the surface of the fungal cell [83]. Later, these metal ions are reduced through enzymatic reduction, which leads to the formation of nanoparticles. The cell-wall sugars also play a major role in the reduction of metal ions [82]. Figure 6 represents a schematic illustration of the possible mechanism involved in the intracellular mycosynthesis of AgNPs.

### 3.3. Mechanism Involved in Phytosynthesis of AgNPs

Several studies have been performed on the elucidation of the mechanism involved in phytosynthesis of metallic nanoparticles, and various theories have been proposed, but the actual mechanism behind the phytosynthesis of nanoparticles is still unknown [84]. However, the common hypothetical mechanism for phytosynthesis mainly involves reducing agents, stabilizing agents, and solvent medium. The phytochemicals present in the plant extract play a dual role as reducing and stabilizing agent in the synthesis of any metal nanoparticle including AgNPs [49,85]. Due to the complex nature of plant extract and the huge number of phytochemicals that are present, it is difficult to identify a specific compound that acts as the reducing and stabilizing agent in the synthesis of nanoparticles. To date, the role of various phytochemicals such as polyphenols (flavonoids, phenolic acid, and terpenoids), organic acid, and proteins have been proposed in the phytosynthesis of metal nanoparticles [86]. Such phytochemicals perform bio-reduction of aqueous metal ions to form a respective zerovalent metal, which further leads to the agglomeration of metal atoms to respective metal nanoparticles [87].

There are some studies suggested that similar mechanisms for phytosynthesis of AgNPs using various plants such as *Pelargonium graveolens* (geranium) [88] and *Azadirachta indica* (neem) [89]. The presence of proteins and other secondary metabolites was reported in the extract of different plants used for the synthesis of AgNPs. Furthermore, it was proposed that terpenoid is responsible for the reduction of silver ions. Moreover, it was also demonstrated that proteins present in the leaf extract of geranium play an important role in the stabilization of AgNPs by capping them [88]. Ahmad et al. [90] proposed that free hydrogen released during keto-enol conversion of flavonoids (luteolin and rosmarinic acid) are mainly responsible for the reduction of metal ions to respective nanoparticles.

**Figure 7:**
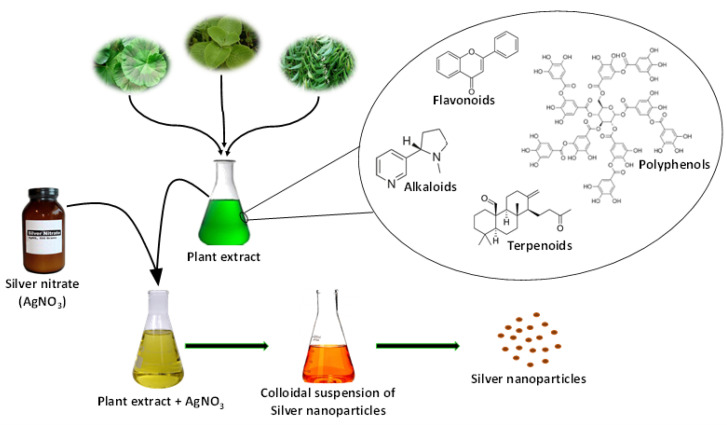
General mechanism involved in the phytosynthesis of AgNPs.

In another study, it was suggested that the reducing sugars present in the leaf extract of *Azadirachta indica* might be responsible for the reduction of silver ions to AgNPs [89]. Moreover, various other compounds such as aromatic amine, amide (I) group, secondary alcohols, and phenolic groups have been proposed for phytosynthesis of AgNPs using *Coleus aromaticus* leaf extract [91]. In addition, the possible role of other phytochemicals, such as various phenolic acids (gallic acid, caffeic acid, ellagic acid, etc.), terpenoids, proteins, and organic acids, etc., in the synthesis of various metal nanoparticles has been critically reviewed [86]. Additionally, it was also observed that the roots of alfalfa have the capability of absorbing silver as AgNPs. An electron microscopic analysis demonstrated the accumulation of silver atoms inside the alfalfa plant tissues, where they underwent nucleation and subsequently formed nanoparticles [92]. Figure 7 shows a schematic representation of the general mechanism involved in the phytosynthesis of AgNPs.

From the above discussion, it can be suggested that different metabolites present in plant extract are responsible for the reduction of metal ions into respective nanoparticles and their capping. In fact, all such capping agents have been found to have the capability of selective binding to different types of facets on nanoparticles, leading to a change in their properties, such as specific surface free energies and their area proportions [93]. Therefore, it is believed that due to capping, nanoparticles can perform several important functions, such as prevention of agglomeration, reduction in toxicity, and enhancement of antimicrobial activity. In addition, there are reports that suggest the enhanced reactivity and catalytic activity of different nanoparticles. Moreover, it was also reported that capping molecules can improve the binding ability of AgNPs on the bacterial cells [94,95]. Similarly, it was reported that the phytochemicals of antimicrobial nature that act as capping agents in phytosynthesis have been found to enhance the antimicrobial efficacy of AgNPs [55].

## 4. Purifications of Nanoparticles

As far as the biogenic synthesis of nanoparticles is concerned, it can be achieved both intracellularly and extracellularly [96]. Some of the biological systems, when exposed to an aqueous solution of metal ions, form nanoparticles intracellularly. Therefore, in such cases, extraction and purification of nanoparticles is essentially required. On the contrary, most biological systems reduce the aqueous metal ions extracellularly, and hence, there is no requirement for the extraction of nanoparticles, but purification is required to remove the impurities present in the form of unreacted components and residues. To date, different techniques have been proposed for the extraction and purification of biogenically synthesized nanoparticles. These techniques mainly include simple filtration, gel filtration, membrane filtration, simple and ultra-centrifugation, electrophoresis, chromatographic methods, chemical or biochemical purification, dialysis, etc. [41,97,98,99,100]. Among these techniques, centrifugation techniques are commonly used for the purification of biogenic AgNPs.

In this context, John et al. [101] demonstrated the intracellular synthesis of AgNPs using a *Pseudomonas* strain. The extraction of the thus synthesized AgNPs was performed by centrifugation of bacterial biomass at 5000 rpm for 30 min. Furthermore, the pellet obtained was suspended in deionized H_2_O followed by ultrasonication at a pulse rate of 6 V at the intervals of 30 s for ten cycles. After sonication, the solution was again centrifuged at 5000 rpm for 30 min, and the supernatant was loaded on a Sephadex G-50 resin equilibrated in 10 mM Tris buffer (pH 7.0). It is required to remove debris and proteins. Finally, the AgNPs were extracted from the buffered solution by adding three volumes of isopropanol because isopropyl alcohol is known to dissolve a wide range of non-polar compounds and evaporate quickly when compared with ethanol. In the end, the mixture was kept in an orbital shaker overnight for evaporation of isopropanol to obtain a pure powder of AgNPs. In another study, Netala et al. [102] demonstrated the extracellular biosynthesis of AgNPs using aqueous callus extract of *Gymnema sylvestre*, and these AgNPs were separated by centrifugation of the solution at 15,000 rpm for 15 min. Furthermore, AgNPs were dispersed in water and purified by repeated centrifugation (five times) to remove the unused callus extract. In another study, Datkhile et al. [103] reported the biogenic synthesis of AgNPs using leaves extract of *Nathophodytes foetida.* The AgNPs were further purified by centrifugation at 10,000 rpm for 30 min and washing with double-distilled water, followed by redispersion of the pellet in deionized water. A similar method of centrifugation (9000 rpm; 20 min; 10 °C) was suggested by Mohanta et al. [25] for the purification of AgNPs synthesized from the extract of *Protium serratum*.

Moreover, Gurunathan et al. [104] performed the extracellular synthesis of AgNPs from *Escherichia coli*. For the purification, the colloidal solution containing AgNPs was washed 5–6 times with deionized water, followed by centrifugation at 10,000 rpm for 15 min. Then the obtained pellet was resuspended in distilled water to remove the remaining unconverted silver ions. Furthermore, this dispersion containing biogenic AgNPs was transferred to a dialysis tube with a 12,000 molecular weight cutoff. Later, the obtained AgNPs were resuspended in 1 mL of HEPES buffer (20 mM, pH 7.4) supplemented with sucrose to reach a density of 2.5 g/mL, and a gradient was made according to proposed standard methods. Finally, the AgNPs were purified by density gradient centrifugation using ultracentrifugation at 200,000 rpm at 4 °C for 16 h. From the above reports, it is clear that centrifugation can be effectively used for the purification of biogenic AgNPs, as it helps to remove untreated ions and other impurities. 

## 5. Multiple Applications of bio-AgNPs in Different Fields

**Figure 8:**
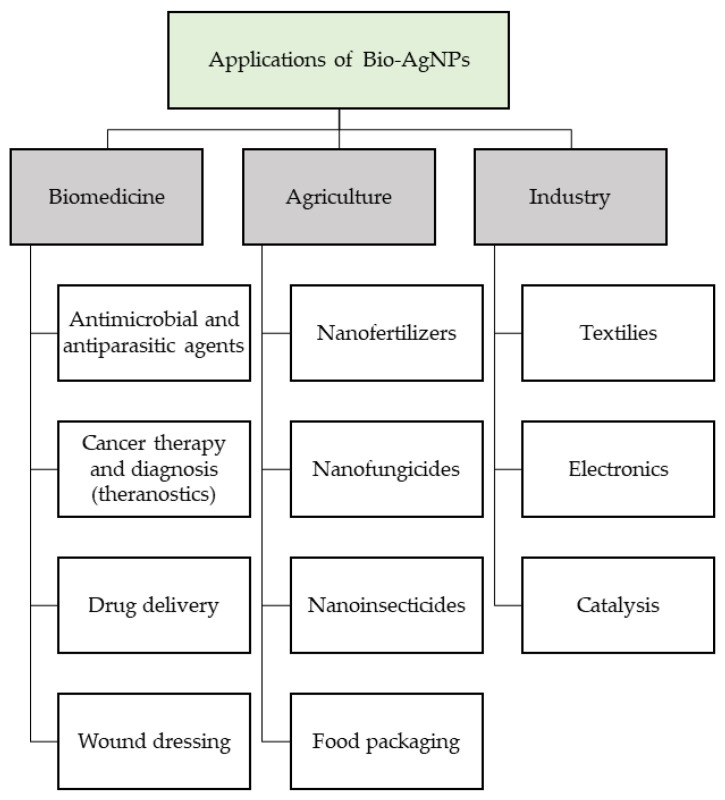
Multiple applications of bio-AgNPs in different fields.

Recent advances in the field of nanotechnology have led to the development of different inorganic and organic nanomaterials. Biogenic AgNPs have already demonstrated multiple applications in many sectors, such as electronics, biomedicine, cosmetics, textile industries, crop protection and growth promotion, food packaging, and biofuel industries [105,106,107]. In recent years, biogenic AgNPs (bio-AgNPs) have attracted the considerable attention of scientists because of their widespread applications in different fields of human life due to their unique properties compared with bulk materials (Figure 8).

Bio-AgNPs have a large active surface area, small size, diverse shapes, biocompatibility, stability, high solubility, and yield without aggregation [50,108,109,110]. The biocompatibility and stability of biogenic nanoparticles may be related to their natural surface coating, which adds some further functionality to the nanoparticles [111,112,113,114]. The widespread applications of bio-AgNPs should also be considered from the ecological and economical point of view, as the biosynthesis process is eco-friendly, easy to perform, and inexpensive [115,116].

### 5.1. Biomedical Applications of Biogenic AgNPs

Nowadays, the applications of various fields of conventional biological sciences, along with innovative and efficient nanotechnological tools for the production of green-synthesized AgNPs, have provided a new alternative strategy and perspective for the prevention and treatment of various diseases [117]. To date, many reports are available that demonstrate the antibacterial, antiviral, antifungal, antiprotozoal, antiparasitic, and anticancer efficacy of bio-AgNPs [9,118,119,120,121,122,123,124,125].

#### 5.1.1. Antibacterial and Antifungal Activities

The antimicrobial nature of AgNPs is the most exploited characteristic of nanosilver in the medical field. Due to the increasing antibiotic resistance of bacteria and the ineffectiveness of conventional therapeutics, there is an urgent need to develop new agents to combat existing pathogens in general and new and emerging pathogens in particular. Nanoparticles can be used as alternative and highly effective antimicrobial agents due to their high surface area, chemical reduction properties, and surface reactivity [9,126,127,128]. There are a number of reports on bio-AgNPs synthesized by bacteria, fungi, and plants that have demonstrated antimicrobial potential against pathogenic bacteria and fungi (Table 3).

Overall, bio-AgNPs show a stronger inhibitory effect on Gram-negative than Gram-positive bacteria. This difference in efficacy may be due to the variance in thickness of the cell wall present in these bacteria [9,141,144,145,146]. Bio-AgNPs were also found to effectively inhibit biofilm formation in Gram-positive (*Stapylococcus aureus*) and Gram-negative (*Escherichia coli*) bacteria [147]. In addition, bio-AgNPs were reported to be active against various human pathogenic fungi such as *Trichophyton mentagrophytes, T. rubrum, T. tonsurans, T. violaceum, Malassezia furfur*, and *Candida* species [148,149,150,151,152,153,154] and also displayed the antibiofilm activity against *C. albicans*, C. *glabrata*, and *C. tropicalis* [147,153].

The antimicrobial efficacies of bio-AgNPs depend upon their physicochemical properties and the biological features of the target pathogens. As far as the mechanism of antimicrobial action of bio-AgNPs is concerned, it can be summarized as follows: (i) electrostatic attraction of bio-AgNPs to cell wall or membrane of microbes, (ii) penetration into the cells, (iii) interaction with biomolecules and intracellular structures, and (iv) free-radical and ROS generation [155]. The changes in cell membrane permeability can cause loss or leakage of intracellular contents, such as ions, proteins, or cellular energy reservoir (ATP), leading to the death of the cell [156]. Bio-AgNPs also affect the function of membrane-bound enzymes such as those in the respiratory chain [157,158] and cause loss of DNA replication and subsequent inactivation of the ribosomal subunit, leading to inhibition of protein synthesis [159]. There are various reports that demonstrate the mechanism of action of bio-AgNPs. For example, *Juniperus excelsa* extract was used for the biosynthesis of small AgNPs (around 16–24 nm) with an extremely large surface area, which showed an ability to bind to the membrane, resulting in the lysis of bacterial cells [160]. In another example, nanoparticles synthesized from walnut extract exhibited a significant antimicrobial activity against Gram-negative (*Escherichia coli* and *Pseudomonas aeruginosa*) and Gram-positive (*Bacillus subtilis*) bacteria. These bio-AgNPs induced bacterial cell permeability by the disruption of cell membrane integrity, either directly or as a consequence of the antimicrobial activity exhibited by such nanostructures [161,162]. Additionally, bio-AgNPs synthesized from *Bacillus cereus* (MT193718) indicated significant antibacterial activity against methicillin-resistant *Staphylococcus aureus* (MRSA) and multidrug-resistant (MRD) *K. pneumoniae*, with a zone of inhibition of 17 and 18 mm at a concentration of 1000 µg/mL and minimum inhibitory concentration (MIC) of 15.6 and 62.5 µg/mL, respectively. Moreover, these bio-AgNPs were found to be compatible with red blood cells at a concentration of 31.5 µg/mL, with no clumping of erythrocytes, which is important to their application as a safe therapeutic agent against multidrug-resistant bacteria [114]. Micro-morphological changes were observed in *C. albicans* cells after treatment with bio-AgNPs synthesized from *Citrus limetta* peel extract. Bio-AgNPs at 10.7 μg/mL caused the cell blebs and a thick exudate deposition around the cell, indicating the leakage of intracellular substances. In another study, although AgNPs produced by *F. oxysporum* showed no significant inhibitory effect on biofilm cells of *C. albicans*, their use in combination with fluconazole caused a significant FLC dose-dependent decrease in viability of FLC-resistant *C. albicans* [163].

#### 5.1.2. Antiviral Activity

The bio-AgNPs have been mainly evaluated for their antimicrobial potential against bacteria and fungi. However, their potential against several types of viruses, including human immunodeficiency virus (HIV), hepatitis B virus (HBV), herpes simplex virus (HSV), respiratory syncytial virus (RSV), and monkey pox virus has also been realized [164,165,166]. The antiviral activity of bio-AgNPs synthesized from marine actinomycete *Nocardiopsis alba* was evaluated on embryonated chicken eggs infected with Newcastle viral disease (NDV), which causes acute respiratory disease and depression. These bio-AgNPs coated with protein derived from the negatively charged carboxylate groups demonstrated promising antiviral activity [167]. In another study, bio-AgNPs synthesized from *Panax ginseng* roots showed antiviral activity against influenza A virus (IAV). The authors reported that the antiviral activity was not only a consequence of the intrinsic effects of bio-AgNPs but also due to the anti-influenza activity of secondary metabolite of plant adsorbed onto the surface of the nanoparticles [168]. Based on these studies, it can be suggested that bio-AgNPs can also be used for the treatment of various viral diseases, including acute respiratory syndromes [169]. Although the existing reports showed promising antiviral activity of bio-AgNPs, more studies are still required to prove the use of bio-AgNPs against novel and emerging viruses such as SARS-CoV-2 [112,170,171].

#### 5.1.3. Antiprotozoal Activity

Biogenic AgNPs are considered as efficient agents against the protozoan parasites responsible for causing zoonoses in humans—namely, *Leishmania amazonensis* and *Toxoplasma gondii* [121,172]. *Leishmania* causes zoonosis called American Cutaneous Leishmaniasis (ACL) which is known to be difficult to treat with available chemotherapy. Biogenic AgNPs synthesized from *Fusarium oxysporum* were found to be a potential agent for the treatment of *L. amazonensis*, in both promastigote and amastigote forms. The bio-AgNPs caused promastigote death, leading to apoptosis-like events due to an increased production of reactive oxygen species (ROS), loss of mitochondrial integrity, phosphatidylserine exposure, and damage of their membrane. Bio-AgNPs reduced the percentage of infected macrophages and the amount of amastigotes per macrophage; consequently, the amount of promastigotes recovered. This biocidal effect of bio-AgNPs was accompanied by a decrease in the ROS level in infected macrophages; thus, bio-AgNPs act on the immunomodulatory ability of infected macrophages, reducing infection without inducing the synthesis of inflammatory mediators. However, the authors suggested further in vivo investigations for a possible bio-AgNP treatment for ACL [121]. In another study, Machado et al. [172] claimed that bio-AgNPs synthesized from *F. oxysporum* can be used as a therapeutic alternative for toxoplasmosis caused by *T. gondii*. They revealed that bio-AgNPs effectively reduced adherence, infection, and proliferation of tachyzoites in HeLa cells at concentrations of 3 and 6 µM when compared with a conventional drug. Bio-AgNPs at the concentrations tested were not able to induce ROS production in HeLa cells infected with *T. gondii* [172].

#### 5.1.4. Anticancer Activity and Theranostics

Biogenic AgNPs may also be an eco-friendly and biocompatible alternative to conventional anticancer drugs [173]. There are various reports concerning the inhibitory effects of bio-AgNPs on many cancer cell lines (e.g., MCF-7 breast cancer, PC-3 prostate cancer, A549 lung cancer, KB oral cancer, PA1 ovarian cancer) [9,123,142,174,175,176,177,178]. The proposed mechanism for anticancer activity of biogenic AgNPs is apoptosis caused through caspase-dependent and mitochondria-dependent pathways induced by nanoparticles [179,180]. It was reported that B16 melanoma cancer cells treated with bio-AgNPs synthesized from *Olax scandens* leaf extract had undergone sub-G1 arrest, which might be the reason for apoptosis induction [181]. Anticancer activity of bio-AgNPs was attributed to the generation of ROS. An increased cellular ROS (O^2•−^, H_2_O_2_) influences the signal transduction pathways that play an important role in apoptosis activation [181]. Studies have shown the upregulation of p53 protein in the lysate of B16 melanoma cancer cells treated with bio-AgNPs. It is well established that the bio-AgNPs treatment ultimately triggers p53 upregulation via apoptosis pathway activation, leading to the cell death. Cancer cells treated with bio-AgNPs have upregulated expression of cleaved/active caspase-3, and silver ions released by bio-AgNPs are the main cause of caspase-3 activation and ultimate oxidative stress [179,181,182,183]. Moreover, the acidic environment of the tumor was assumed to be the cause of phyto-constituents being released from bio-AgNPs, enhancing the potential anticancer activity of bio- AgNPs [181]. Biogenic AgNPs were found to possess antiangiogenic property with the inhibition of VEGF-induced cell proliferation [179,184,185], which provides another proof that they can be exploited for the development of potent cancer therapeutic agents.

An in vitro analysis also showed that the bio-AgNPs synthesized from actinobacterial strain SF23 demonstrated higher cytotoxicity against MCF-7 breast cancer cells than RAW 264.7 macrophages. In addition, these AgNPs stimulated cancer cells to release a greater amount of ROS than macrophages, which can be correlated with higher cancer cell mortality. These studies indicate that the use of bio-AgNPs may be an effective anticancer therapy itself, while activation of macrophages may additionally involve defense mechanism against tumors [9]. The induced apoptosis, cytotoxicity, and anticancer activity of bio-AgNPs synthesized from *Artemisia turcomanica* leaf extract against gastric cancer cell lines, in a dose- and time-dependent manner, were reported by Mousavi et al. [180]. Bio-AgNPs synthesized using extract of *Sesbania grandiflora* L. showed cytotoxic effect against the MCF-7 cancer cell line, causing loss of cell membrane integrity, oxidative stress, and apoptosis [186]. Some reports also proved that bio-AgNPs possess higher anticancer properties than those produced by physical and chemical methods [180]. A lower concentration of phyto-synthesized AgNPs from *Artemisia marschalliana* was able to inhibit growth of human gastric (AGS) cells when compared with the commercial agents [187]. Moreover, bio-AgNPs are believed to increase the efficiency of a particular anticancer drug by targeting it specifically to particular cancer cells (targeted drug delivery), which will ultimately decrease the dosage of the drug and minimize the side effects [188]. Bio-AgNPs combined with cancer drug doxorubicin showed a significantly elevated anticancer activity on the B16F10 cell line, as compared with the drug attached to chemically synthesized nanoparticles [189].

It is believed that in the future, bio-AgNPs will be promising entities administered for the diagnosis of cancer due to their self-fluorescence ability [179], but to date, the evidence has been insufficient. Researchers reported the self-fluorescence ability of bio-AgNPs synthesized from the methanolic extract of *Olax scandens*. Cancer cells treated with bio-AgNPs coated with fluorescent molecules from plant extract emitted bright-red fluorescence, indicating the internalization of these nanoparticles by cells. Such observations were not recorded in untreated cells or in cells treated with chemically synthesized AgNPs [179,181].

#### 5.1.5. Antidiabetic Activity

Green synthesized AgNPs are also reported as antidiabetic agents; for example, bio-AgNPs synthesized from *Holoptelea integrifolia* were found to possess antidiabetic activity. Although various extracts of *H. integrifolia* have shown antidiabetic activity by the inhibition of the ATP-sensitive potassium channels in the pancreatic beta cells mechanism, the AgNPs synthesized from this plant showed an enhanced effect. This activity of bio-AgNPs increased in a dose dependent manner. A 60.08 ± 3.38% of α-amylase inhibition was observed by employing 25 μL of tested AgNPs, while in case of plant extract and standard (acarbose) a 45.88 ± 3.44 and 72.22 ± 4.3% inhibition was observed. At a higher concentration of 100 μL, the percentage of enzyme inhibition in the presence of AgNPs, extract, and acarbose was 86.66 ± 5.03, 71.28 ± 4.33, and 95.01 ± 5.41%, respectively [189]. The studies concerning the antidiabetic properties of bio-AgNPs are scanty, and therefore, more research concerning efficacy and toxicity are needed to draw conclusions.

### 5.2. Antioxidant Activity

It is well known that antioxidants protect cells against free radicals. An antioxidant stops oxidation by neutralization of produced free radicals, as a result of which it undergoes oxidation [190]. Antioxidant activity of bio-AgNPs refers to the formation of non-reactive stable radicals through an inhibition of the oxidation of molecules by preventing the initiation step of the oxidative chain reaction. The antioxidant potential of bio-AgNPs depends on the properties of various molecules deposited on their surface. The capping of reduced silver by secondary metabolites may augment the involvement of synthesized AgNPs towards the antioxidant property [189].

Bio-AgNPs synthesized from the aqueous extract of *Holoptelea integrifolia* leaves exhibited significant antioxidant activity (51.49 ± 3.33% for 2,2-diphenyl-1- picrylhydrazyl-hydrate (DPPH), 41.18 ± 2.27 for metal chelating (MC), and 74.59 ± 3.08% for nitric oxide (NO) assays) at the highest tested concentration of NPs. In DPPH, MC, and NO assays, a concentration-dependent effect was noticed for these AgNPs. The lower values in the case of DPPH and MC assays when compared with the NO assay was attributed to the secondary metabolites that were present in low concentrations of *H. integrifolia* extract. It was interpreted that in the case of the MC assay, secondary metabolites had a capping mechanism with silver metal, so they were not free; hence, low MC properties were acceptable. Moreover, it was claimed that different biomolecules may affect different antioxidant properties when estimated using different antioxidant assays but that they can help in reducing oxidative stress in cells. The authors also claimed that biogenic nanosilver could serve as a free radical scavenger, possibly acting as a primary antioxidant. Bio-AgNPs may be a good alternative to synthetic antioxidants having adverse health effects, such as butylated hydroxyl toluene, butylated hydroxyl anisole, and propyl gallate [189]. Similarly, Ibrahim et al. [190] reported effective antioxidant properties of bio-AgNPs synthesized by *Bacillus cereus* using DPPH and 2,2′-azino-bis-3-ethylbenzothiazoline-6-sulfonic acid (ABTS) assays.

### 5.3. Application of Biogenic AgNPs in Agriculture

Crop pathogens reduce the yield and quality of agricultural production [191]. Various strategies are used in the management of plant diseases, but some of them impose a serious negative impact on the environment [192]. By providing new agrochemicals and tools for delivering active compounds, nanotechnology offers the possibility of reducing and optimizing the use of conventional products, such as toxic pesticides [193].

#### 5.3.1. Plant Protection

The antifungal activity of biogenic AgNPs against important phytopathogens (e.g., *Fusarium oxysporum, F. tricinctum, Botrytis cinerea*, *Penicillium expansum*, *Aspergillus niger*, *Alternaria* sp., and *Rhizopus* sp.) has been reported by many authors [119,194,195,196], but mainly in vitro. The bio-AgNPs synthesized by *Serratia* sp. BHU-S4 were examined under in vitro conditions for antifungal activity against *Bipolaris sorokiniana,* the spot blotch pathogen of wheat. Bio-AgNPs accounted for total inhibition of conidial germination. The biocontrol potential of AgNPs against *B. sorokiniana* was also confirmed by in vivo assay. *B. sorokiniana* after nanoparticle treatment did not develop spot blotch on wheat leaves [197]. In another study, bio-AgNPs fabricated using *Nigrospora oryzae,* the corn grain contaminant, exhibited dose-dependent antifungal activity against *Fusarium* spp., an important plant pathogenic fungus [135]. Bio-AgNPs synthesized from leaves extract of *Acalypha indica* at a concentration of 15 mg/10 µl showed strong antifungal activity against all tested fungi (*Alternaria alternata, Botrytis cinerea, Curvularia lunata, Rhizoctonia solani,* *Sclerotinia sclerotiorum*, and *Macrophomina phaseolina*), which was evident from the inhibition zones of growth in the range of 18–23 mm [198]. In addition, the nematicidal activity of bio-AgNP was confirmed against plant parasitic nematode *Meloidogyne incognita*. Encouragingly, bio-AgNPs significantly reduced the nematode activity, mortality, egg hatching, and movement of larvae. In another experiment, instead of chemical nematicide products, bio-AgNPs synthesized using *Acalypha wilkesiana* aqueous extract could be recommended to manage the plant-parasitic nematode, as a simple, stable, and cost-effective way of keeping the environment safe [199]. It is expected that the application of bio-AgNPs at low concentrations will be eco-friendly and will decrease farm management costs. Based on the above discussion and the potential role of bio-AgNPs against plant pathogens, it is evident that these may be used as an alternative solution for controlling microbial pathogens affecting plant growth instead of using synthetic chemicals. However, it is necessary to determine the exact mechanism of bio-AgNPs action in the fungal cell and their impact on the environment and human beings [200].

#### 5.3.2. Plant Growth Promotion

Nanoparticles can increase the vigor of the crops to withstand the impact of pests and diseases [193]. There are many reports indicating that, in low concentrations, bio-AgNPs have a positive effect on seed germination and the promotion of plant growth [201]. For example, AgNPs synthesized by a green method using *Berberis lycium* Royle extract had the capacity to improve crop growth and yield [202]. In another study, bio-AgNPs synthesized by using the aqueous extract of *Euphorbia helioscopia* leaves, when applied on seeds and as foliar sprays, had a positive impact on the morphology of seed oil, enzymes, and fatty acid content of sunflower [203]. Bio-AgNPs synthesized from a marine endophytic fungus, *Fusarium equiseti*, displayed positive effects on wet weight, shoot length, root length, chlorophyll, and carotenoid content, even at a lower concentration of 5 ppm. These results suggest that bio-AgNPs could be used as a nanofertilizer after performing further toxicity studies under field conditions [204]. Zhang et al. [205] compared chemically (chem-AgNPs) and biologically (bio-AgNPs) synthesized AgNPs. It was found that chem-AgNPs had strong antibacterial activity against *E. coli*, while bio-AgNPs exhibited long-term antibacterial effects. In addition, chem-AgNPs showed toxic effects on cucumber plants by inducing over-generation of ROS, thus resulting in the upregulation of malondialdehyde (MDA) and Zn content and the downregulation of antioxidant capacity, carotenoid, globulin, and Mo content, while biogenic AgNPs significantly promoted photosynthesis in cucumber. Moreover, bio-AgNPs enhanced the protein content and stimulated the upregulation of Mn and downregulation of Al. Nevertheless, after treatment with bio-AgNPs, the downregulation of Mo and the upregulation of Al indicated minimal toxicity to cucumber plants. Overall, bio-AgNPs when compared with chem-AgNPs exhibited limited toxic effects to cucumber plants. Bio-AgNPs have been proven to be biocompatible, well-dispersed, and because of their high efficiency and low toxicity, could be used as nanopesticides in agriculture [205].

### 5.4. Food Packaging

Bacterial and fungal contaminants can spoil food by degrading its quality and causing an unpleasant taste. In addition, microbial contamination of food poses a threat to human health [206]. Bio-AgNPs incorporated with biopolymers may find applications as protective agents in food storage and preservation [207]. Biodegradable nanocomposite films can be developed by incorporating AgNPs into the gelatin biopolymer matrix for food packaging applications. Bio-AgNPs were synthesized by using industrial food waste cassava tuber peels which showed significant antimicrobial activity, depicting their capability of being used in a wide range of applications in food and pharmaceutical industries. Moreover, the integration of AgNPs improved the mechanical and barrier properties of gelatin film. The nanocomposite films increased the shelf life of sapodilla fruits, which indicated the potential of the films for the food packaging industry, as a way of extending the shelf life of packaged food by up to 12–15 days [208]. Antioxidant and antimicrobial AgNPs synthesized by using *Mussaenda frondosa* leaves extract were loaded into gelatin/chitosan composite films that enhanced the shelf life of vegetables and fruits. From an ecological point of view, the use of biodegradable AgNPs films may reduce synthetic plastics consumption and environmental harm and may promote healthy foods [209]. In another study, *Myxobacteria virescens* synthesized AgNPs were impregnated into fruit wrapping paper, which increased the shelf life of apples up to 15 days as compared with the non-impregnated wrapper [210].

### 5.5. Smart Nanotextiles

Nanotechnology is already having a huge impact on the textile industry. Nanocoating of the surface of textiles or footwear is one approach for the production of highly active surfaces with UV blocking, antimicrobial, and self-cleaning properties [211,212]. Biologically synthesized AgNPs from *Azadirachta indica* leaf extract were used in sock fabrics (nylon and cotton), being antimicrobial in nature. These nanosilver-coated socks were found to have a highly active surface with antimicrobial and self-cleaning properties. The anti-odor, non-toxic, and durable properties of the textiles were also confirmed. Therefore, bio-AgNPs can be effective in reducing foot-borne infections and can be used for various applications, including applications in healthcare and medicine [211]. The inclusion of bio-AgNPs into cotton fibers improved their thermal stability and elongation properties. Moreover, fibers embedded with nanoparticles exhibited remarkable antimicrobial activity against *E. coli*. Therefore, these fibers have great potential for utilization, e.g., in burn/wound dressings, as well as in the fabrication of antibacterial finishing of textiles [213]. It was reported, that AgNPs adhered to or inserted into textile fibers exhibited an effective antibacterial activity against *Bacillus subtilis* by release of Ag^+^ ions [214]. Multifunctional cotton fabric was also prepared through a simple method based on the coating of the fabric with AgNPs and low surface energy silane. The prepared fabric simultaneously gained functionalities, such as water repellency, antibacterial activity, and UV-blocking [215]. The UV-protection abilities of wool were highly improved with the application of bio-AgNPs. AgNPs were in situ synthesized by using natural compounds and biomolecules of plant extracts (naphthoquinones, phenolics/flavonoids, polyphenols) as reducing or stabilizing agents and were simultaneously deposited on wool fabric for coloration, UV protection, and antioxidant properties. The antioxidant activity of material mainly depends on reducing/stabilizing compounds of nanoparticles from plant extract [216]. The AgNPs synthesis was carried out using aqueous extract of lemon leaves (*Citrus limon*), which acts as a reducing agent and encapsulating cage for the AgNPs. By incorporating nanoscale silver into textiles, the manufacturers can make materials that use a small amount of silver for elimination of the microbes present on the surface of the clothing material. Such AgNPs that have relatively large surface area available are ideally suited for the effective control of bacteria and molds and can help to prevent spoilage from microbial growth in damp areas [217]. Multifunctional viscose fibers were successfully prepared by simultaneous dyeing and incorporation of bio-AgNPs that were fabricated through a green approach using compounds extracted from peanut red skin. The obtained fabric exhibited UV protection, antimicrobial activity, and coloration, as well as antioxidant activity [218]. Cotton fabric with multi-protective properties was achieved by the environmentally friendly in situ synthesis of AgNPs. The plant material from food waste (e.g., green tea, avocado seed, and pomegranate peel) and invasive plant material (e.g., Japanese knotweed rhizome, goldenrod flowers, and staghorn sumac fruit) were used as reducing agents for the formation of AgNPs directly on cotton fabrics, which provided excellent protection against UV radiation as well as against *Escherichia coli* and *Staphylococcus aureus* bacteria [219]. 

### 5.6. Catalytic Activity

Biogenic AgNPs act as catalysts for the degradation of organic dyes (methylene blue, methyl orange, congo red tartrazine, carmoisine, and brilliant blue FCF) and other toxic compounds such as 4-nitrophenol to non-toxic compounds [220,221,222], which reduces environmental pollution [221]. Bio-AgNPs synthesized from aqueous *Prosopis juliflora* leaves extract were evaluated for catalytic activity against azo dyes such a methylene blue (MB) and congo red (CR) that resulted in its effective degradation of toxic compounds in a short span of time. The reduction of dyes became faster in the presence of bio-AgNPs than NaBH_4_. In addition, MB was reduced faster in the presence of bio-AgNPs when compared with CR. Moreover, the reduction of 4-nitrophenol to non-toxic 4-aminophenol was investigated with aqueous NaBH_4_ along with AgNPs that acted as a catalyst for the reaction. Although the reduction of 4-NP into 4-AP with NaBH_4_ can be possible, it is limited by kinetic barriers due to the difference in the thermodynamic potential of an electron donor (NaBH_4_) and acceptor (4-NP) that decreases the feasibility of the reaction. In this case, the use of AgNPs as nanocatalysts facilitated the electron relay from donor to acceptor [190,220]. Similarly, bio-AgNPs synthesized from the fruit extract of *Viburnum opulus* L. were found to have catalytic ability in the degradation of tartrazine, carmoisine, and brilliant blue FCF dyes by NaBH_4_ as reducing agent. The results demonstrated remarkable activity against all the investigated dyes, being an outstanding catalyst for the degradation of brilliant blue FCF. The authors concluded that bio-AgNPs can be used as powerful tools for reducing environmental pollution from organic dyes [221]. The reduction of 4-NP to 4-AP and the degradation of methyl orange (MO) and MB in the presence of sodium borohydride and catalyzed by bio-AgNPs synthesized using aqueous extract of fruit peel (*Citrus macroptera*) and bacteria (*Bacillus cereus*) were also reported recently [190,222]. Considering the above facts, it can be inferred that bio-AgNPs can be used as catalysts for the degradation of dyes.

### 5.7. AgNPs in Sensor Development

In addition to various biological and catalytic properties, the unique opto-electronic properties possessed by silver nanoparticles have opened the door for their use in sensing applications. Although this field is not fully explored, there are a few reports available on the application of biogenic AgNPs in the development of nanosensors. Nanosensors are used for different purposes, including the detection of heavy metal ions and the degradation of complex compounds, dyes, pollutants, and other contaminants that have extremely low detection limits [223]. Actually, heavy metals and other contaminants are generated in the environment as a result of both human and industrial activities, and these are found to be toxic to almost all living forms in higher concentrations. Therefore, detection and degradation of such compounds are necessary. Certain conventional methods are already available for this purpose, but considering their limitations, newer, highly effective approaches need to be developed. However, in view of the noteworthy applications of nanotechnology in general and biogenic nanoparticles in particular, it is believed that biogenic AgNPs can be effectively used in the development of sensors that can be promisingly used in the detection of various contaminants and also in helping with the degradation of toxic compounds in the environment. Some available reports on biogenic AgNPs-based sensors are discussed briefly here.

As mentioned above, the presence of heavy metal ions in the environment is a global health concern. Therefore, their removal is required to minimize environmental pollution. In this context, Hoyos et al. [224] developed a nanosensor using AgNPs synthesized from an aqueous extract of *Camellia sinensis* (green tea) having a mean diameter of 7 nm. Furthermore, these green synthesized AgNPs were found to exhibit good sensing properties towards Cu^2+^ and Pb^2+^ ions in aqueous solutions. This metal ions-sensing ability of the biogenic AgNPs was recorded with help of UV-Vis spectrophotometry (SPR analyses) and fluorescence spectroscopy. Recently, Al-Thabaiti and Khan [225] synthesized biogenic AgNPs by anthocyanin obtained from red rose petals. Furthermore, the thus synthesized AgNPs were used as a sensor for the detection of bromothymol blue and also as a catalyst for the oxidative degradation of bromothymol blue in the presence of sodium borohydride, hydrogen peroxide, and sunlight. In another study, Hussain et al. [226] developed nano-based sensors using biogenic AgNPs synthesized from an aqueous extract of clove (*Syzygium aromaticum*). These AgNPs were used as a colorimetric sensor for the detection of trace amounts of vinclozolin (fungicide) by UV-Vis spectroscopy for the first time. The authors claimed that these biogenic AgNP-based sensors were found to be very sensitive, simple, green, economically viable, and highly selective in colorimetric detection of vinclozolin. 

In another study, Tagad et al. [227] used biogenically synthesized AgNPs from locust bean gum (LBG) polysaccharides in the development of optical fiber-based hydrogen peroxide (H_2_O_2_) sensors. H_2_O_2_ is commonly used in water treatment plants and other various industries for disinfection and cleaning microcircuits. The access concentration of H_2_O_2_ induces many kinds of cellular damage; therefore, the determination of H_2_O_2_ level is of great importance. In addition, the concentration of H_2_O_2_ needs to be monitored in food and pharmaceutical industries and clinical laboratories. Therefore, the above discussed H_2_O_2_ sensor can be effectively used for this purpose. Similarly, Wani et al. [223] reviewed the role of biogenically synthesized AgNPs in the development of colorimetric and electrochemical sensors, which can be promisingly used for the detection of various pollutants, such as heavy metal ions, H_2_O_2_, NH_3_, nitrite ions, sulfide ions, kanamycin, nitrobenzene, and biomolecules such as nucleic acids, aminoamides, etc. Furthermore, the authors concluded that biogenic AgNPs can be used as a promising material for the optical and electrochemical sensing of various types of pollutants in water and soil. Moreover, these nano-based sensors could be very effective for the detection of various biomolecules. 

## 6. Biodistribution of AgNPs

The biogenic AgNPs not only helps in the design of safer nanomaterials but also assists in providing a better understanding of health and safety concerns. The biomaterial-based methods do not require the use of hazardous chemicals; therefore, useful products can be generated quickly at a reasonable scale and in an eco-friendly manner [228]. There is a lack of appropriate and standard characterization methodologies that might be used for research evaluating the toxicity of AgNPs to compare the results of different investigations employing similar NPs. Biogenic nanoparticles are harmless, environmentally benign, and contribute to a greener approach. As discussed before, bio-AgNPs can also be used in a variety of research and technology sectors. However, there are some concerns that must be addressed, such as choice of synthesis method, biodistribution, and toxicity issues [228,229]. Biosynthesis utilizing plant extracts for antimicrobial applications, biocidal characteristics, and cytotoxicity depends on physiochemical parameters such as size, concentration, and coating—all of which have been discussed in recent studies [229,230]. AgNPs can cause inflammation and oxidative stress at the site of exposure. Furthermore, they have the ability to penetrate a variety of biological barriers and reach systemic circulation. AgNPs that have been administered intravenously are immediately accessible in circulation. AgNPs are subsequently transported to numerous organs, where they produce organ-specific pathogenic consequences. It is unclear whether the effects found in distant organs are due to the direct impact of translocated AgNPs or particle-induced inflammatory and oxidative stress responses at the exposure site. Ferdous et al. [231] demonstrated AgNPs translocation, accumulation, and toxicity to numerous organs following various modes of exposure, such as inhalation, instillation, and the oral, cutaneous, and intravenous route. Particle size, coating, route, and length of exposure, dosages, and endpoint measurement time all impact the effects on local and distant organs.

**Figure 9:**
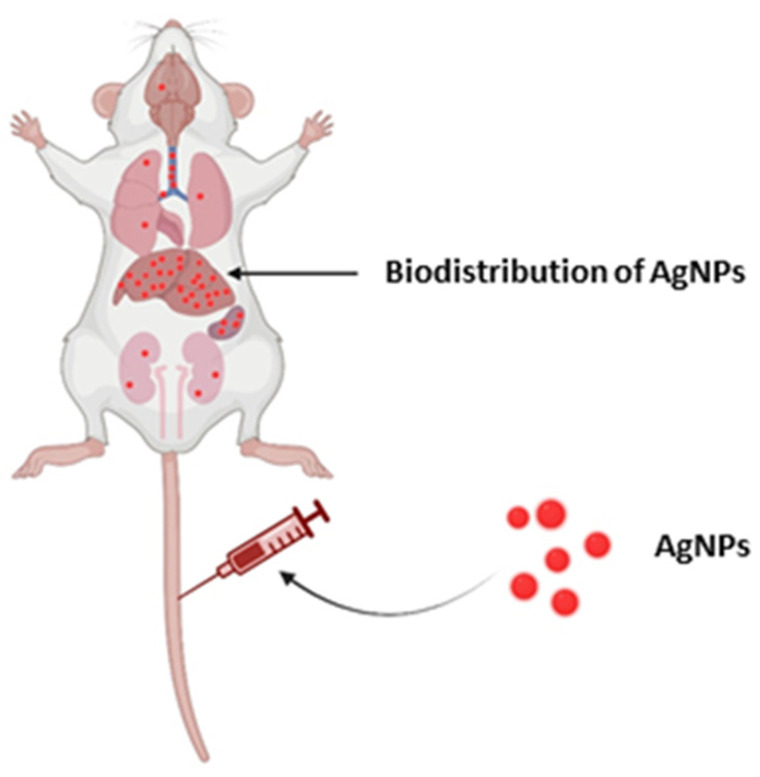
Biodistribution of AgNPs in rat organs.

Subchronic inhalation or injection of AgNPs demonstrated the uptake of silver ions and nanoparticles in the blood and subsequent distribution to all major organs and tissues, including the liver, kidneys, testes, ovaries, olfactory bulb, and brain. However, the level of accumulation of AgNPs in different organs was found to be different [232] (Figure 9). Human bodies are vulnerable to AgNPs when such AgNPs are consumed, inhaled, or absorbed through the skin because of the large surface area-to-volume ratio, and their penetrating potential is greatly increased, allowing them to penetrate the circulatory system and even to translocate freely within the human body system [233,234]. Yang et al. [235] revealed that the size, surface functionalization, and concentration of AgNPs all have a role in in vivo dispersion. Furthermore, AgNPs’ inherent chemical composition resulted in erratic biodistribution and hazardous profiles, which received some attention. The biodistribution, toxicity kinetics, and genotoxicity variations in murine animals were studied using AgNPs.

AgNPs were predominantly deposited in the mononuclear phagocyte system (MPS), such as the liver and spleen. The AgNPs were accumulated in organs, such as the heart, lung, kidney, and other organs. AgNPs were also found in greater concentrations in the circulation and feces. Over the course of two months, measurements of mouse body and organ mass, hematological and biochemistry evaluations, and histological investigations revealed a little harmful effect of AgNPs. AgNPs caused higher alterations in gene expression related to oxidative stress, apoptosis, and ion transport, according to RT qPCR results. The chemical properties of NPs were demonstrated by their findings. Male Wistar rats were given AgNPs (20 and 200 nm) intravenously at a dosage of 5 mg/kg of body weight. Following injection, biological samples were collected after 24 h, 7 days, and 28 days. The concentration of silver in tissue was determined using inductively coupled plasma-mass spectrometry (ICP-MS) and transmission electron microscopy after AgNPs were translocated from the blood to the main organs (TEM). The liver contained the highest amount of silver after 24 h. After 7 days, a considerable quantity of silver was found in the lungs and spleen. The concentration of silver in the kidneys and brain increased during the experiment, reaching a peak after 28 days. Furthermore, the highest concentration of AgNPs was identified in the urine 1 day after the injection, stayed high for 14 days, and then dropped. The final results showed that the fecal level of silver in rats peaked two days after AgNPs treatment and subsequently gradually declined [236].

Zande et al. [237] investigated AgNPs biodistribution in rats. They presented the findings of a 28-day oral exposure trial in rats that were given either 20 nm noncoated AgNPs or 15 nm PVP-coated AgNPs (90 mg/kg body weight or AgNO_3_ (Ag) = 9 mg/kg body weight) or a carrier solution. After dissecting on day 29 and a 1- or 8-week wash-out period, AgNPs were found in all of the organs tested, with the greatest amounts in the liver and spleen for all treatments. The quantity of Ag^+^ in the silver nanoparticle solution was significantly associated with silver concentrations in the organs, showing that predominantly Ag^+^, and to a lesser degree AgNPs, went through the intestines in silver nanoparticle-exposed rats. After 8 weeks, silver was removed from most organs in all groups, but not from the brain or testis. Using single particle inductively coupled plasma mass spectrometry, AgNPs were detected in silver nanoparticle-exposed rats, but remarkably also in AgNO_3_ exposed rats, hereby demonstrating the formation of nanoparticles from Ag^+^ in vivo that are probably composed of silver salts. The silver exposure did not cause hepatotoxicity or immunotoxicity, according to biochemical indicators and antibody levels in the blood, lymphocyte proliferation and cytokine production, and NK-cell activity. Finally, it appears that oral exposure to AgNPs is quite comparable with oral exposure to silver salts. However, the long-term effects of AgNPs in vivo and long-term preservation of AgNPs are unknown [238].

## 7. Toxicity of Biogenic AgNPs

Nowadays, the nanomaterials, especially nanoparticles (NPs), are being widely used; therefore, their fabrication and application have resulted in public awareness of their toxicity and impact on the environment [15,238]. In addition to the wide range of applications of AgNPs, it is very crucial to take account of in vivo-associated toxicity and immunoreactivity. Worldwide reports on the toxic effects of NPs that were aimed at identifying the targets and mechanisms of their harmful effects were carried out mainly using different cell culture models, including cancer cell lines [9,239]. However, the effect of bio-AgNPs on the latter was discussed earlier. In contrast, few studies on the patterns of NPs transport, accumulation, degradation, and elimination using animal models have been performed [55]. For this reason, in recent years, the number of nanotoxicology studies that have investigated the biological pathways affected by nanoparticles and induced toxic effects has increased substantially [240]. There is no doubt that deeper knowledge of the mechanisms of bio-AgNPs’ effect on living organisms will discover new areas of their application in the near future.

Overall, it is claimed that chemically synthesized AgNPs have high in vivo cyto- and genotoxicity as compared with biogenic AgNPs, proposing the latter to be less toxic and biocompatible for potential applications [179,241].

Cytotoxicity effects of biogenic AgNPs against eukaryotic cells have been reported by many authors [9,23,123]. Researchers around the world provided new approaches for a more comprehensive understanding of the mechanism of NP-induced toxicity [55]. It is claimed that cytotoxicity of AgNPs is largely mediated by the action of Ag^+^ ions released from nanoparticles [242] that interact with cells and intracellular macromolecules such as proteins and DNA [243]. Moreover, other authors suggested that one of the potential mechanism of bio-AgNPs cytotoxicity is the generation of ROS and superoxide synthesis following reduction of oxygen by electron from electron transport chain on the mitochondrial surface [9,55,244]. The generated ROS thus lead to oxidative damage of cellular contents including DNA [242], proteins, and lipids [230,242], and consequently to cell death [242] or to apoptosis [182,183]. The study by Wypij et al. [9] showed that AgNPs-stimulated MCF-7 cells released a greater amount of ROS than RAW 264.7 macrophages, which correlated with higher cancer cell mortality. The authors suggested that AgNPs-induced cell death can be partially mediated by ROS production. However, their previous studies are in contradiction to the above-mentioned report. In this case the higher sensitivity of the macrophages to the AgNPs was explained as a ROS-dependent phenomenon, which may be related to the scavenger receptor pathway and the scavenger function of macrophages that increase their sensitivity to the effects of nanoparticles [123]. Moreover, AgNPs themselves may have a devastating effect on the protective antioxidant enzymes, resulting in cellular damage by oxidizing vital biomolecules, subsequently leading to cell death [244].

In addition, ions released from AgNPs may increase the cytotoxic effect of AgNPs by inducing cascades that lead to intracellular toxicity, defined as the “Trojan horse effect” [55]. Akter et al. [230] underlines that the mechanism generation of free radicals occurs after interaction of NPs with cellular components, especially with mitochondria. According to El-Naggar and coauthors [244], apoptosis could be activated through mitochondrial dysfunction, which potentially inhibits the proliferation of cells. Wypij and coauthors [9] estimated the reactive oxygen species (ROS) level in the presence of biosynthesized AgNPs on MCF-7 human breast cancer cell line and murine macrophage cell line RAW 264.7. However, some eukaryotic cells are more prone to nanomaterials, especially AgNPs, than to others due to the presence of both the released Ag ions and AgNPs [15,240]. Hamida and coauthors [245] showed that AgNPs induced the greatest toxic effects against HepG2 cells when compared with MCF-7 and Caco-2. The authors emphasized that such effect could have been due to charge and the type of biomolecules surrounding AgNPs.

The toxicity of nanoparticles to the cells also depends on their size, shape, and coating/capping agent, and their concentration and surface composition. Many of these conclusions come from the study of the effects of AgNPs on cancer cells [15,122,243]. According to Recordati and coauthors [246], smaller AgNPs due to their larger surface area-to-volume ratio have a faster rate of releasing of the silver ion (Ag^+^), hence an increased bioavailability, enhanced distribution, and toxicity of Ag compared with larger NPs. The significant impact of AgNPs size on their biological activity has been reported by many authors [243,247,248]. Ashajyothi and Chandrakanth [240] studied animal toxicity using 11–75 nm biogenic AgNPs (bio-AgNPs) in the male Wistar rat. It was also reported that the cytotoxicity of bio-AgNPs against normal and cancer cell lines is dose-dependent. In many cases, nanoparticles at low concentrations do not cause significant toxicity [9,123,247,249]. The shape of the AgNPs might influence the cellular uptake mechanism, which in turn modulates the cytotoxicity [230]. An important aspect in biogenic nanoparticle toxicity is their coating. The biogenic nanoparticles can be synthesized by using organisms or by products of their metabolism. Therefore, these nanoparticles are capped with biomolecules of natural origin that can improve stability and determine their biological activity and cytotoxicity [250].

## 8. Conclusions

Nanotechnology is playing a pivotal role in the day-to-day life of human beings due to its wide-range of applications. Nanoparticles are the tools of nanotechnology that can be synthesized by physical, chemical, and biological methods. The latter is green, sustainable, rapid, and economically viable process of synthesis. These biogenic nanoparticles have demonstrated multiple applications, such as in the biomedical field, agriculture, as catalysts, in textiles, as biosensors, and so on. As far as the biomedical sector is concerned, nanomaterials in general and AgNPs in particular are considered as magic bullets that can revolutionize this sector by the development of newer nano-based antimicrobials or by their application in drug delivery. Biogenic AgNPs have been reported to have better biological efficacies over physically and chemically synthesized nanoparticles due to their biocompatibility and enhanced biological properties. Moreover, also in agriculture, biogenically synthesized nanoparticles have been found to enhance the growth of crop plants and may protect them from the attack of pathogens and pests. The AgNPs can also be used for gene transfer in plants. Various efforts have been made to elucidate the exact mechanisms involved in the biogenic synthesis of AgNPs, but unfortunately, to date no such mechanism is known.

## Figures and Tables

**Table 1:** Important physical, chemical, and photochemical methods used for the synthesis and stabilization of AgNPs [15].

Method	Precursor	Reducing Agent	Stabilizing Agent	Size (nm)
Chemical Methods				
Chemical reduction	AgNO_3_	N,N′-dimethylformamide	-	<25
Chemical reduction	AgNO_3_	Sodium borohydrate	Surfactin (a lipopeptide biosurfactant)	3–28
Chemical reduction	AgNO_3_	Trisodium citrate (initial) +SFS (secondary)	Trisodium citrate	<50
Chemical reduction	AgNO_3_	Trisodium citrate	Trisodium citrate	30–60
Chemical reduction	AgNO_3_	Ascorbic acid	-	200–650
Chemical reduction	AgNO_3_	Sodium borohydrate	Dodecanoic acid	~7
Chemical reduction	AgNO_3_	Paraffin	Oleylamine	10–14
Chemical reduction (thermal)	AgNO_3_	Dextrose	Polyvinyl pyrrolidone	22 ± 4.7
Chemical reduction (thermal)	AgNO_3_	Hydrazine	-	2–10
Chemical reduction (oxidation of glucose)	AgNO_3_	Glucose	Gluconic acid	40–80
Chemical reduction (polyol process)	AgNO_3_	Ethylene glycol	Polyvinyl pyrrolidone	5–25
Chemical reduction (polyol process)	AgNO_3_	Ethylene glycol	Polyvinyl pyrrolidone	50–115
Chemical reduction (microemulsion)	AgNO_3_	Hydrazine hydrate	Aerosol-OT	2–5
Chemical reduction (microemulsion)	AgNO_3_	Hydrazine hydrate	Aerosol-OT	<1.6
Electrochemical (polyol process)	AgNO_3_	Electrolysis cathode: titanium anode: Pt	Polyvinyl pyrrolidone	~11
Chemical reduction (Tollen)	AgNO_3_	m-Hydroxy benzaldehyde	Sodium formaldehyde sulphoxylate	15–260
Physical Methods				
Physical synthesis	Ag wires	Electrical arc discharge, water	-	14–27
Physical synthesis	AgNO_3_	Electrical arc discharge	Sodium citrate	2–5
Photochemical Methods				
Photochemical reduction (pulse radiolysis)	AgClO_4_	Ethylene glycol	-	17–70
Photochemical reduction (microwave radiation)	AgNO_3_	Ethylene glycol	Polyvinyl pyrrolidone	5–10
Photochemical Reduction (photoreduction)	AgNO_3_	UV light	-	4–10
Photochemical reduction (X-ray radiolysis)	Ag_2_SO_4_	X-Ray	-	~28
Photochemical reduction (X-ray radiolysis)	AgNO_3_	Carboxymethylated chitosan, UV	Carboxymethylated chitosan	2–8

**Table 2:** List of biological agents used for the synthesis of AgNPs.

Biological Agent	Type	Mode of Synthesis	Size (nm)	Shape	References
*Urospora* sp.	Seaweed	Extracellular	20–30	Spherical	[30]
*Aspergillus flavus*	Fungus	Extracellular	33.5	Spherical	[21]
*Capparis spinosa*	Plant	Extracellular	5–30	Spherical	[31]
*Protium serratum*	Plant	Extracellular	74.56 ± 0.46	Spherical	[25]
*Trichoderma longibrachiatum*	Fungus	Extracellular	10	Spherical	[32]
*Caesalpinia ferrera* (Tul.) Maritus	Plant	Extracellular	30–50	Spheroidal	[33]
*Trichoderma harzianum*	Fungus	Extracellular	50–80	-	[22]
*Zea mays*	Plant	Extracellular	25	Spherical	[26]
*Torreya nucifera*	Plant	Extracellular	10–125	Spherical	[34]
*Bacillus siamensis*	Endophytic Bacteria	Extracellular	25–50	Spherical	[17]
*Aspergillus fumigatus*	Fungus	Extracellular	84.4	Spherical	[35]
*Talaromyces purpurogenus*	Fungus	Extracellular	4–41	Spherical, hexagonal, rod-shaped, and triangular-	[23]
*Eucalyptus corymbia*	Plant	Extracellular	18–20	Spherical	[27]
*Equisetum arvense*	Plant	Extracellular	18–20	-	[36]
*Cucumis prophetarum*	Plant	Extracellular	30–50	Irregular granulated, ellipsoidal	[37]
*Leptolyngbya* sp. WUC 59	Cyanobacteria	Extracellular	20–35	Spherical	[20]
*Lysiloma acapulsensis*	Plant	Extracellular	1.2–62	Spherical and quasi-spherical	[28]
*Shewanella* sp. ARY1	Bacteria	Extracellular	38	Spherical	[18]
*Fusarium scirpi*	Fungus	Extracellular	2–20	Quasi-spherical	[24]
*Synechocystis* sp.	Microalgae	Extracellular	10–100	-	[38]
*Citrobacter freundii*	Bacteria	Extracellular	15–30	Spherical	[19]
*Aspergillus sydowii*	Fungus	Extracellular	1–24	Spherical	[39]

**Table 3:** Selected examples of biogenic AgNPs and their antimicrobial activity Key: MDR; multidrug-resistant.

Organism	Activity	Target	References
Bacteria			
*Pilimelia columellifera* subsp. *pallida* (SF23, C9)	antifungal	*Malassezia furfur, Trichophyton rubrum, Candida albicans*, *C. tropicalis*	[129]
*Streptomyces* sp. OSIP1 and OSNP14	antibacterial	*Staphylococcus aureus Bacillus subtilis, Proteus mirabilis Escherichia coli, Pseudomonas aeruginosa*	[130]
*Bacillus cereus*	antibacterial	*Escherichia fergusonii, Streptococcus mutans*	[131]
*Pseudomonas rhodesiae*	antibacterial	*Dickeya dadantii*	[132]
*Alcaligenes* sp.	antibacterial and antifungal	*Bacillus* sp., *Escherichia coli*, *Klebsiella pneumonia, Pseudomonas aeruginosa*, *Staphylococcus aureus, Candida albicans*	[133]
*Bacillus brevis*	antibacterial	MDR *Staphylococcus aureus, Salmonella typhi*	[134]
Fungi			
*Nigrospora oryzae*	antifungal	*Fusarium* spp.	[135]
*Alternaria* sp.	antibacterial	*Bacillus subtilis, Staphylococcus aureus, Escherichia coli, Serratia marcescens*	[136]
* Phomopsis helianthi *	antibacterial	* Escherichia coli, Pseudomonas aeruginosa *	[137]
*Colletotrichum* sp.	antibacterial	* Escherichia coli, Bacillus subtilis, Staphylococcus aureus, Salmonella typhimurium *	[138]
*Aspergillus tubingensis*	antifungal	*Candida albicans, Candida glabrata, Candida parapsilosis*	[139]
*Cladosporium cladosporioides*	antibacterial and antifungal	*Staphylococcus aureus, Staphylococcus epidermis, Bacillus subtilis, Escherichia coli, Candida albicans*	[140]
Plants			
*Juglans regia*	antibacterial	*Escherichia coli*, *Pseudomonas aeruginosa*, *Staphylococcus aureus*	[141]
*Dimocarpus Longan*	antibacterial	*Escherichia coli, Staphylococcus aureus*	[142]
*Eucalyptus camaldulensis*	antifungal	*Candida albicans*	[143]
